# A Machine-Learning-Based Prediction Model for Total Glycoalkaloid Accumulation in Yukon Gold Potatoes

**DOI:** 10.3390/foods14193431

**Published:** 2025-10-07

**Authors:** Saipriya Ramalingam, Diksha Singla, Mainak Pal Chowdhury, Michele Konschuh, Chandra Bhan Singh

**Affiliations:** 1 Advanced Post-Harvest Technology Centre, Lethbridge Polytechnic, Lethbridge, AB T1K 1L6, Canada; 2 Biological Sciences, University of Lethbridge, Lethbridge, AB T1K 3M4, Canada

**Keywords:** potato quality, TGA, hyperspectral imaging

## Abstract

Potatoes are the most extensively cultivated vegetable crop in Canada and rank as the fifth largest primary agricultural commodity. Given their diverse end uses and significant market value, particularly in processed forms, ensuring consistent quality from harvest to consumption is of critical importance. Total glycoalkaloids (TGA) are nitrogen-containing secondary metabolites that are known to accumulate in the tuber as an effect of greening in-field or elsewhere in the supply chain. In this study, 210 Yukon Gold (YG) potatoes were exposed to a constant light source to green over a period of 14 days and sampled in 7-day intervals. The samples were scanned using a short-wave infrared (SWIR) hyperspectral imaging camera in the 900–2500 nm wavelength range. Once individually scanned, pixel-wise spectral data was extracted and averaged for each tuber and matched with its respective ground truth TGA values which were obtained using a High-Performance Liquid Chromatography (HPLC) system. Prediction models using the partial least squares regression technique were developed from the extracted hyperspectral data and reference TGA values. Wavelength selection techniques such as competitive adaptive re-weighted sampling (CARS) and backward elimination (BE) were deployed to reduce the number of contributing wavelengths for practical applications. The best model resulted in a correlation coefficient of cross-validation (R^2^_cv_) of 0.72 with a root mean square error of cross-validation (RMSE_cv_) of 51.50 ppm.

## 1. Introduction

Potato tubers (*Solanum tuberosum* L.) are a globally significant crop, serving as a staple food and a critical source of energy, vitamins and minerals. In Canada, Alberta has emerged as a leading potato-producing province, with Southern Alberta playing a pivotal role in this success [1]. The area’s favorable climate is characterized by hot days and cool nights, combined with high-quality soil and an optimal growing season of 110 to 130 days, creating ideal conditions for potato cultivation. This advantageous environment has enabled Southern Alberta to become a powerhouse in potato production, contributing significantly to both the provincial and national economies.

Among the various potato cultivars, the Yukon Gold variety holds a special place due to its unique characteristics and historical significance. Developed in the 1960s at the University of Guelph in Ontario, Canada, Yukon Gold (YG) was the first Canadian-bred potato variety to be promoted, packaged and marketed with its name directly on the packaging. It is renowned for its smooth, thin skin and vibrant yellow flesh, offering a creamy texture and rich flavor that have made it a favorite among consumers and chefs alike [2]. Used widely in boiling, baking and frying, YG adds to its widespread market appeal by being one of the best fresh market potatoes.

However, during post-harvest handling and storage, potatoes are prone to quality deterioration and chemical changes that can compromise both food safety and marketability [3]. One such concern is the accumulation of total glycoalkaloids (TGAs), toxic secondary metabolites primarily composed of α-solanine and α-chaconine. While TGAs naturally occur in potatoes, their levels can significantly increase under environmental stressors such as light exposure, leading to greening, which is associated with elevated chlorophyll and TGA content [4]. When consumed in high concentrations, glycoalkaloids pose severe health risks, including gastrointestinal discomfort and neurological effects, necessitating monitoring of their levels in potatoes. As per Health Canada guidelines, the TGA concentration in any potato variety should not exceed 200 mg/kg fresh weight (200 ppm) prior to marketing [5]. Beyond health risks, excessive TGA levels can lead to product rejections in markets, affecting supply chains and producer revenue. Moreover, consumer perception of visible greening and toxicity in potatoes can undermine confidence in the product, even when levels remain within regulatory limits. This highlights the need for a rapid, accurate and a preferably non-destructive method to assess TGA concentrations in post-harvest management systems.

Traditional methods for quantifying TGAs, such as High-Performance Liquid Chromatography (HPLC) and liquid chromatography-mass spectrometry (LC-MS), are highly accurate and reliable [6]. However, these techniques are destructive, time consuming and labor-intensive, making them unsuitable for real-time, large-scale applications in commercial supply chains. To address this gap, non-destructive techniques, particularly those leveraging hyperspectral imaging (HSI) in the short-wave infrared (SWIR) spectrum, have emerged as powerful tools [7].

A combination of digital imaging and spectroscopy, the hyperspectral imaging system is capable of generating a 3D “hypercube”, which has two spatial dimensions and one spectral dimension [8]. Due to its penetrability, the NIR hyperspectral imaging system can be used to capture vital chemical changes in complex food products [9]. Therefore, extraction of meaningful data is possible through the application of mathematical and statistical approaches, commonly known as chemometrics [10]. Some of the common approaches used to develop predictive models include regression techniques such as partial least squares regression (PLSR); Support Vector Machine Regression (SVMR), Random Forest and Artificial Neural Networks (ANN) [11]. Such approaches are followed by the application of effective data-mining techniques for more effective prediction such as competitive adaptive reweighted sampling (CARS), backward elimination (BE), Iteratively Retaining Informative Variables (IRIV), etc. [12]

This study focuses on developing a predictive model for TGA levels in Yukon Gold potatoes exposed to light for up to 14 days using SWIR hyperspectral imaging and machine-learning tools.

Therefore, the objectives of this study were as follows:

1. Explore the capabilities of deploying SWIR hyperspectral imaging to non-destructively estimate TGA levels in YG potatoes.
2. Apply chemometric and regression techniques such as PLSR and SVMR to develop predictive models.
3. Study the effectiveness of feature selection tools (CARS and IRIV) to improve model performance.

## 2. Materials and Methods

### 2.1. Chemicals and Reagents

The α-solanine and α-chaconine standards were purchased from INDOFINE Chemical Company, Inc. (Hillsborough, NJ, USA). The HPLC-grade solvents acetonitrile and glacial acetic acid and other analytical-grade chemicals and solvents were procured from VWR International Co. (Mississauga, ON, USA). Milli-Q water was used to prepare all necessary reagents in this study.

### 2.2. Sample Collection and Preparation

The Yukon Gold (YG) potatoes used in this experiment were procured from a local market in Southern Alberta, Canada. Potato samples were first washed with water to remove surface contaminants. Tuber samples that had visible surface defects such as bruising or other forms of mechanical damage were promptly excluded from the study. Batches of potatoes totaling 210 healthy YG tubers were exposed to a constant LED light source (2 × 4 LED Panel light, 3300 lumens, Liteline, Ontario, Canada) for a total of 14 days. Samples were collected in 7-day intervals where Day 0 samples were considered as the control, while light-exposed samples were collected on Day 7 and 14. The controlled light exposure was chosen to mimic typical greening conditions that occur during post-harvest storage, ensuring repeatability and reproducibility of results. While real-world conditions may vary in intensity and duration, the chosen setup provided a standardized baseline that enabled model training under controlled stress. Once collected, the samples were scanned under the SWIR hyperspectral camera tuned to the SWIR range (900–2500 nm).

### 2.3. Hyperspectral System and Image Acquisition

The SWIR hyperspectral image acquisition system consists of key components such as the illumination source (150 W Halogen Lamp), a motion device with a translation stage and sample tray and a computer with Hyperspec III Software (version v3.1.5-RC1, Ground Operations) suite installed (Headwall Photonics NIR, Series M, Bolton, Massachusetts, USA). Each tuber sample was imaged in reflectance mode in the SWIR range (900–2500 nm). To ensure image quality, the frame period and exposure were set at 30 ms and 13 ms, respectively, and maintained throughout the course of the experiment. These settings were selected based on preliminary scans, which balanced spectral resolution and signal-to-noise ratio for biological samples. Prior to image acquisition, the system was turned on for 30 min and set to a power level of 70% maximum (105 W). This step ensured even illumination and good image quality across all samples. Tubers were placed on the non-reflective surface of the 40 × 20 cm motion device. A fluoropolymer white calibration tile (99% reflectance) (Spectralon, Labsphere, North Sutton, NH, USA) was used to obtain white reference, while the camera shutter was kept closed (0% reflectance) during dark reference collection. Once white and dark references were captured, the samples were scanned at a velocity of 13.147 mm/s in the push-broom mode, resulting in sharp images of the tuber. Tubers that appeared progressively greener upon exposure to light were scanned for their total glycoalkaloid content. A 3D- hypercube was generated consisting of 464 × 368 × 169 pixels, with 169 being the number of spectral channels or wavelength bands. The images were captured at regular intervals of 9.527 nm starting at 901.121 nm through 2501.676 nm. Background correction and data normalization (from pixels to reflectance values) were automatically performed by the Hyperspec III software. Once scanned, the potatoes were diced evenly into small cubes and placed into numbered sample-holder tins. The tins were weighed with and without sample and placed into the Taxi-Dry Freeze-Dryer system (Freezedry Specialties Inc., Model ARA1800 R.V.T, Princeton, MN, USA), which was maintained at 25 mm Hg vacuum for an average of 3 days. Upon the removal of moisture, the tubers were immediately transferred to individual zip-lock bags and sealed. A laboratory-grade grinder was used to prepare a fine powder of the freeze-dried samples, which were stored until further analysis at 4 °C.

### 2.4. Extraction and Purification of Total Glycoalkaloids (TGA)

Total glycoalkaloids (TGA) were extracted by mixing 10 g of the freeze-dried powdered sample with an extraction solution containing Milli-Q water, glacial acetic acid and sodium hydrogen bisulfite. The mixture was vortexed, sonicated for 1 h and centrifuged at 4400 rpm for 30 min. The same procedure was repeated thrice on the resultant pellet, followed by pooling the supernatants together and storing them at 4 °C. Extract purification was performed using solid-phase extraction (SPE) with Hypersil™ C18 column, which was conditioned with acetonitrile and extraction solution. The extract was passed through the column, washed with 15% acetonitrile and eluted using a liquid chromatography (LC) mobile phase. The final volume was adjusted to 5 mL, filtered (0.45 µm PTFE) and transferred to HPLC vials for analysis.

### 2.5. HPLC Analysis of TGA

The quantification of TGAs was performed using a HPLC system (Perkin Elmer, LC 300, Woodbridge, ON, USA). The LC300 HPLC system comprises an HPLC quaternary pump, autosampler, column oven and photodiode array (PDA) detector. Separation was achieved using an Epic C18 column (250 mm × 4.6 mm, 5 µm particle size) maintained at 25 °C. Chromatographic data were processed using Simplicity Chrom software (version 1.6), PerkinElmer, Connecticut, USA. Isocratic elution was carried out using a mobile phase comprising 60% acetonitrile, 30% Milli-Q water and 10% 0.01 M sodium phosphate buffer (pH 7.2) at a flow rate of 1 mL/min. The injection volume was set to 10 µL, and the run time was 10 min. The eluent was monitored at a wavelength of 202 nm. TGA concentrations were calculated using standard calibration curves prepared with α-solanine and α-chaconine standards and expressed in parts per million (ppm).

### 2.6. Spectral Pre-Processing and Data Analysis

The autocorrected .HDR spectral images were first converted to a .Mat file (MATLAB (MathWorks Inc., R2023b, Natick, MA, USA) for ease of processing. As no dead pixels were noticed, filter operations were not performed on the spectral data. The mask of each tuber was prepared using Otsu’s thresholding technique which separates the foreground of the image from its background. This marks the region where the tuber sits as ‘1’ while the background is kept as ‘0’ [13]. Next, the Region of Interest (ROI) was generated upon applying the mask across all the wavebands in the hypercube. The mean spectrum of all samples imaged in the SWIR range (900–2500 nm) was obtained by averaging the pixel intensities of each spectral image. This resulted in an n × 169 matrix, where ‘n’ is the number of tuber samples and 169 is the number of wavebands the SWIR-HSI system is capable of scanning. Following optimal data collection, its conditioning was performed by pre-processing of spectral data, which is one of the most important steps.

### 2.7. Dimensionality Reduction

The hyperspectral images obtained comprise 169 wavelengths and are multicollinear, which means that they contain overlapping information. This makes data bulky, redundant and of higher dimension, rendering it less useful and larger in volume [14]. Since the model aims to be deployed at large-scale commercial applications in the future, image processing speed must match the pace of acquisition. Therefore, choosing the most relevant features within the dataset to build and train the machine-learning model is essential; and this process is commonly known as feature selection. Providing essential wavelengths (reduced number of variables) will not only speed up computational efficiency but also make predictions more precise.

#### 2.7.1. CARS and Backward Elimination

In this study, competitive adaptive re-weighted sampling (CARS) was performed, followed by backward elimination (BE). Previous studies have shown that the CARS algorithm proved to be an effective tool in reducing the feature space by iteratively removing redundant wavelengths [15]. Briefly, the feature-selection process begins with a Monte Carlo selection algorithm where ‘N’ random subsets of samples are drawn from the full dataset. PLSR is then performed on the subset which generates the absolute value of the regression coefficient (|β|), based on which the contributing wavelengths are indexed. This operation is repeated N times and wavelengths with small regression coefficients are eliminated during the exponentially decaying function (EDF) and adaptive reweighted sampling (ARS) step. At the end of the selection process, only the best-performing variables (wavelengths) are retained. Here, PLSR (5-fold cross- validation) on the reduced feature space was performed and its respective RMSE_cv_ was noted. The output of CARS was the feature space that yielded the lowest RMSE_cv_. To further reduce the number of predictive features, the CARS output was subject to a backward elimination (BE) operation. This greedy feature elimination technique identifies and retains only those wavelengths that contribute significantly to outcome prediction. Starting with the full set of variables, the model is trained and evaluated. The least significant feature is selected and removed, after which the model is retrained, eliminating more features based on performance. This iterative elimination process continues until a decline in model performance, or a stop criterion is encountered [16,17].

#### 2.7.2. Iteratively Retaining Informative Variables (IRIV)

Iteratively Retaining Informative Variables (IRIV) is a variable selection technique that uses the Binary Matix Shuffling Filter technique (BMSF) to choose variables of interest. This technique operates by deploying the Mann–Whitney U-test to observe the variation in RMSE_cv_ to evaluate the significance of each variable while selecting features to build a regression model [18]. The variables are first classified one after the other upon inclusion and exclusion, keeping other variables consistently included or excluded. This results in them being classified into categories ranging from highly informative to interfering based on the RMSE_cv_. For example, if the RMSE_cv_ decreases upon the exclusion of a variable, it implies that the variable is uninformative or interfering. On the other hand, if the RMSE_cv_ increases upon the exclusion of the variable, it is considered informative [19]. In this study, the process of IRIV-based selection was followed by backward elimination to remove less informative wavelengths, like the CARS feature reduction process described in Section 2.7.1.

#### 2.7.3. Prediction and Model Metrics

In this study, two regression techniques—partial least square regression (PLSR) and Support Vector Machine Regression (SVMR)—were deployed to analyze multivariate data. PLSR is a recognized statistical technique that uses both Principal Component Analysis (PCA) and multiple regression (MR) [20]. With ‘X’ number of variables being highly collinear in chemometric analysis, PLSR can explain the variance in X and the covariance between X and Y (ground-truth). In a typical PLSR model, X is projected onto a set of orthogonal components based on its respective weight vector known as latent variables (LVs). The ideal number of LVs are chosen based on the lowest observed RMSE_cv_ value.

The SVMR is a supervised machine-learning algorithm that is more suitable for non-linear relationships. Unlike PLSR, where data of a higher dimension is projected onto a lower dimension using latent variables, SVMR uses hyperparameters such as (C, ε and kernel function) to control the learning process [21]. The complexity and model accuracy are determined by the factor “C”, also commonly known as the regularization parameter. “ε” specifies the error tolerated without penalty. In the current study kernel, ‘radial bias function’ (RBF) was chosen, which can identify non-linear relationships. First, multiple SVR models were run using a range of C and ε values using the best features obtained from CARS and IRIV as input. It was observed that too small or too large a value of hyperparameters negatively affected the performance of the model and hence a combination of both values that best represented the dataset was chosen and kept constant across all predictive models.

Model performance was evaluated using the leave-one-out cross-validation (LOOCV) approach. Here, the model is trained using (n-1) observations and tested against the remaining one. This technique is most useful for small datasets as they provide the most unbiased estimate of model metrics [22]. As an additional test of model accuracy, a train:test split (70:30) was performed using MATLAB’s inbuilt *cvpartition* function to mitigate bias. This research assessed the performance of the developed PLSR and SVMR models using the coefficients of determination of cross-validation (*R*^2^*_cv_*), prediction (*R*^2^*_p_*) and calibration (*R*^2^*_c_*). Respectively, the root mean square error values for cross-validation (*RMSE_cv_*), prediction (*RMSE_p_*) and calibration (*RMSE*_c_) were also deduced using the following equations.

(1) $$R_{cv}^{2},\;R_{p}^{2},R_{c}^{2}=1-\left(\frac{\sum_{i=1}^{n}(y_{i\;}{-Y_{i})}^{2}}{{\sum_{i=1}^{n}(y_{i\;}-\hat{Y})}^{2}}\;\right)$$

(2) $${RMSE}_{cv},{RMSE}_{p},{RMSE}_{c}={\sqrt{\frac{1}{n}\;\sum_{i=1\;}^{n}\left(y_{i}-Y_{i}\right)}}^{2}$$

Here, ‘$y_{i\;}$’ represents the actual TGA values in the *i*th sample of the dataset while the predicted TGA values are given by ‘$Y_{i}$’. Mean TGA values are given by ‘$\hat{Y}$’ for ‘n’ number of samples in the complete dataset. High *R^2^_cv, p_* values in conjunction with lower *RMSE_cv,p,c_* were preferred, indicating a strong correlation between the spectral (n × m) and response data matrices (n × 1).

The robustness of the models developed using the PLSR and SVMR approaches were also assessed by calculating their Residual Predictive Deviation (*RPD*) given by the following equation.

(3) $$\mathrm{RPD}=\frac{{SD\;}_{p}}{RMSEp}$$

The *RPD* metric is the ratio of the predictive standard deviation to its root mean square error. This statistical tool is used to measure how well the model can predict compositional data (TGA). If a large root mean square error is calculated against the spread of the compound of interest (standard deviation), the resultant is a low *RPD* value that indicates a weak predictive model. While an *RPD* above 2.5 is preferred, values between 1.4 and 1.8 are considered fair and can be used for assessment.

**Figure 1:**
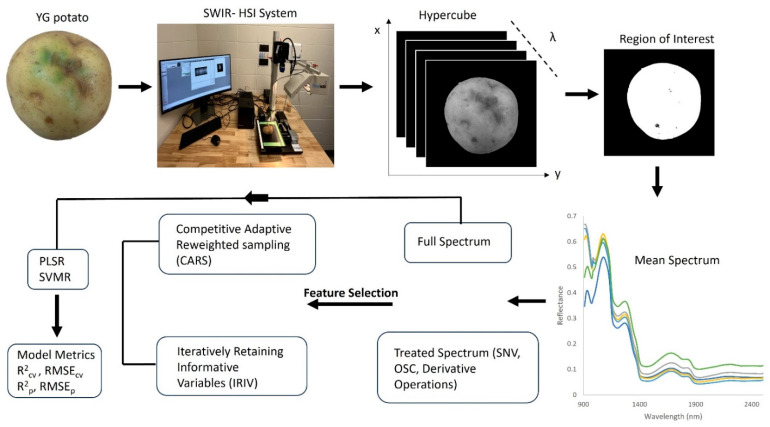
Flow chart of the methodology adopted for the prediction of TGA in YG potatoes indicating mean spectrum from select tubers.

MATLAB was used to develop a script for data extraction from the hyperspectral images, feature selection using CARS and IRIV and model development—PLSR and SVMR. Figure 1 displays the overall methodology used to extract the mean spectral data from each YG hypercube, scanned in the 900–2500 nm wavelength range.

## 3. Results and Discussion

### 3.1. Total Glycoalkaloids in Tuber Samples

**Figure 2:**
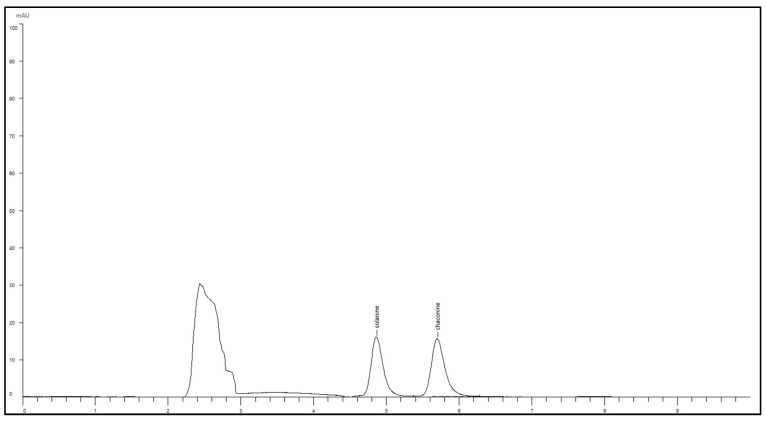
HPLC chromatogram of solanine and chaconine at a concentration of 100 ppm.

The HPLC was able to provide well-resolved peaks for α-solanine and α-chaconine when a standard cocktail of 100 ppm was introduced (Figure 2). Sample retention times remained consistent across all tuber extracts, averaging at 4.86 min and 5.70 min, respectively. A standard graph for both compounds was developed, which was further used to quantify the extent of TGA present in the tuber samples.

Although TGA levels increased from Day 0 (97.13 ppm) to Day 7 (159.56 ppm), a decline was observed on Day 14 (128.41 ppm), indicating a slight non-linear accumulation pattern. This trend may be attributed to the physiological response of the tubers to prolonged light exposure. Initially, light stimulates glycoalkaloid biosynthesis; however, extended exposure may induce photooxidative stress, leading to metabolic shifts such as enzymatic degradation or redistribution of glycoalkaloids within tuber tissues [23]. Furthermore, factors like desiccation, cellular aging, or feedback inhibition mechanisms may contribute to the reduction in TGA levels after their peak.

The results align with previous studies, including Machado et al. [24] and Percival & Dixon [25], who documented fluctuations and overall increases in TGA levels under light exposure. Rymuza et al. [26] also noted significant TGA accumulation across different potato varieties, with light exposure increasing TGA content in a parabolic fashion. The current study corroborates these findings, demonstrating that prolonged light exposure leads to consistent TGA accumulation, peaking after approximately 7 days, as reported by earlier findings.

### 3.2. Spectral Analysis

**Figure 3:**
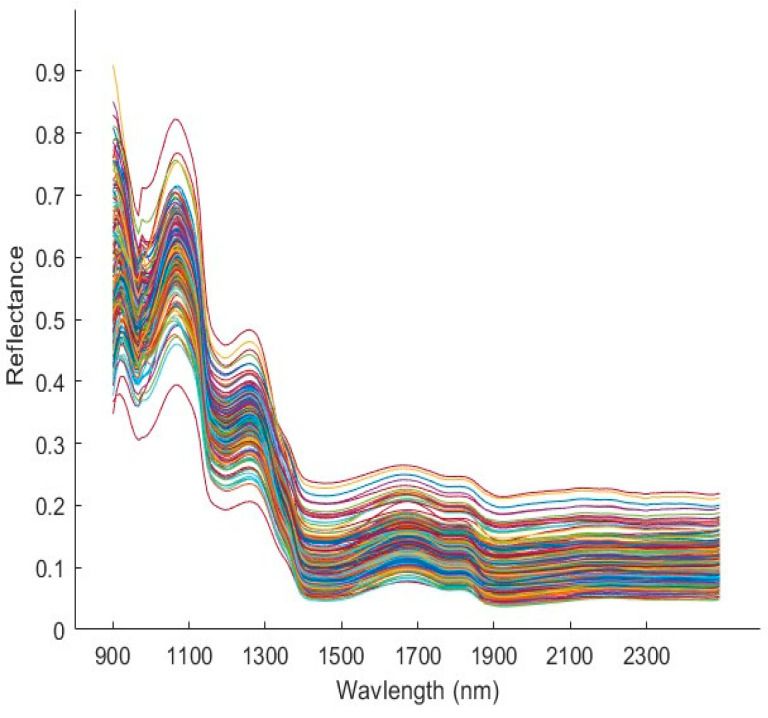
Mean reflectance spectra of all Yukon Gold potato samples scanned in the SWIR range (900–2500 nm), indicated in individual colors.

The spectral curves associated with YG potatoes scanned on the side exposed to light is shown in Figure 3. The uniformity of the spectral curves was maintained throughout the 210 tuber samples which indicated no deviations in illumination and the appropriate collection of white and dark references. The absorption bands observed in the spectral data of biological samples correspond to water content, proteins, lipids, starch, etc. In the case of Yukon Gold potatoes, five significant absorption peaks were observed in the mean spectrum. The peaks at 976, 1431 and 1905 nm correspond to the 3rd overtone of O-H stretching vibration, 1st O-H overtone, and O-H stretching and deformation, respectively [27]. These absorption peaks represent water and carbohydrates in the tubers. The prominent peak at 1214 nm chemically corresponds to the 2nd overtone of C-H stretching vibrations that are commonly associated with the presence of starch. The absorption peak at 1781 nm indicates the 1st vibrational overtone of C-H related to the sugar content of the tuber [28]. The information gained about molecular vibrations (bending, stretching, etc.) of the functional groups provides vital insight into the organic compounds present in the sample. Additionally, peaks related to the glycoalkaloids of interest such as α-solanine and α-chaconine were not observed in the SWIR spectral data. This is likely because these complex steroid-like compounds exist as an intact oligosaccharide (linked to a sugar molecule) [29]. Such occurrences emphasize the importance of using chemometric tools to explain the relationship between total glycoalkaloids and the spectral data obtained.

### 3.3. Full Spectrum Model

The spectral data obtained for all scanned tuber samples were concatenated with their respective ground-truth values for all 169 wavebands. PLSR and SVMR models were run on this dataset upon the application of spectral pre-processing techniques. The LOOCV performance of the PLSR and SVMR models were based on the resultant R^2^_cv_ and RMSE_cv_ values (Table 1). It is evident from the models developed with no spectral pre-processing that PLSR out-performed SVMR. This might be because PLSR is more tuned to handling highly collinear and high dimensional data by projecting the predictors into latent variables (LVs) that explain the maximum covariance between spectral data (X) and predictor values (Y). Results of this study validated the importance of performing pre-processing steps to improve model metrics. The application of derivative techniques, in addition to orthogonal signal correction (OSC), displayed improved performance in the prediction of total glycoalkaloids in YG potatoes. The OSC works by removing variations in the spectral data (X) that do not relate to the predictors (Y), thereby improving the predictive power of the model. It is also known to address light scattering effects, which, in our case, would have been caused by the uneven surface of the tuber itself. Another great tool used to improve the resolution of multivariate data is through the application of derivative techniques. They are highly sensitive to the rate of change in reflectance in spectral data, thereby eliminating background variations. Slightly better performance was observed with the SVMR model over PLSR, when OSC and 1st derivative pre-processing were performed on the full spectrum. It is however a difficult task to generalize the application of pre-processing strategies across different types of datasets. For instance, factors such as sample orientation, unwanted artifacts and dark current influence the choice of pre-processing technique that best improves model performance. Therefore, to reduce computational time and optimize model operation, a reduced feature space is preferred.

### 3.4. Model Development with Feature Wavelengths

#### Variable Selection Using CARS and BE

**Figure 4:**
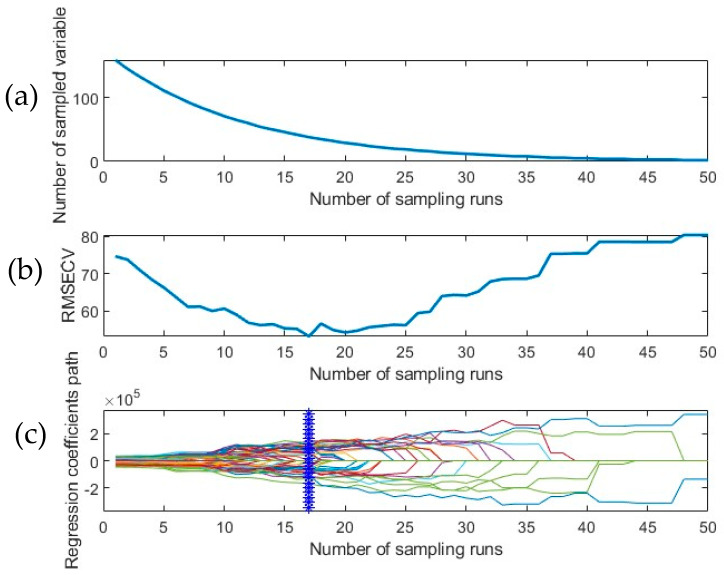
CARS graph presenting the trend followed during (**a**) EDF-based feature elimination in the dataset, (**b**) fluctuations in the root mean square error of cross validation RMSE_cv_ over sampling runs upon a 5-fold CV operation (**c**) path taken by regression coefficients vs. the number of sampling runs.

A large dataspace of 169 channels (full spectrum) increases the time taken to train the model, hence limiting the applicability of the SWIR-HSI system in a field setting. In this study, the CARS algorithm was applied to the full dataset (spectral data with respective ground truth values) across each tuber sample. CARS reduced 169 channels into a small number of characteristic wavelengths which were the most relevant to the input data. The iterative algorithm adjusts the list of selected wavelengths based on an adaptive reweighted sampling (ARS) technique based on the absolute value of the regression coefficient in every PLSR run. Figure 4a depicts the trajectory taken by the EDF to rapidly remove non-informative variables in the initial few iterations, thus reducing the number of wavelengths available for selection. The EDF is followed by ARS, which deploys a competitive mechanism to eliminate wavelengths sequentially. An initial decrease in RMSE_cv_ can be observed in Figure 4b due to the elimination of wavelengths that did not significantly contribute to meaningful information in the developed predictive model. However, after the 17th sampling run, a steady increase in RMSE_cv_ is noted which is indicative of the removal of essential features (wavelengths) that impact the performance of the model. Monitoring the variations that occur in the regression coefficient profile over the course of the simulation is crucial. A subgroup of wavelengths is generated in each iteration because of a 5-fold cross-validation study. The blue vertical line in Figure 4c indicates the point at which the model reaches the lowest 5-fold RMSE_cv_ derived from the best-performing subset of wavelengths selected by CARS. Table 2 illustrates the outcome of applying CARS on the full spectrum along with the best-performing pre-processing technique and the corresponding wavelengths that were selected during this process. PLSR performed on OSC + 1st derivative pre-processed spectra yielded an R^2^_cv_ value of 0.707 and RMSE_cv_ of 52.9 ppm. CARS was able to reduce the feature space to 32 wavelengths from a full spectrum of 169, thereby improving performance and addressing the issue of multi-collinearity. The CARS operation was followed by BE, which resulted in a set of “best-features” that predicted the presence of total glycoalkaloids from the developed dataset (Table 3). It can be observed that the backward elimination algorithm applied in conjunction with different pre-processing techniques significantly reduced the number of contributing wavelengths, thereby retaining the most relevant information required to build the model.

LOOCV performance metrics for the developed SVMR and PLSR models using the CARS + BE selected wavelengths are given in Table 4. The best PLSR model performance was obtained by applying the 1st derivative operation on the dataset that resulted in an R^2^_cv_ value of 0.723 and RMSE_cv_ of 51.50 ppm. A slight improvement in cross-validation results was noticed compared to the results obtained from just the CARS output, indicating that the BE operation was able to successfully remove redundant features. Among the CARS + BE-SVMR models developed with the dataset, OSC + 1st derivative pre-treatment provided the best metric—R^2^_cv_ value of 0.711, RMSE_cv_ of 52.60 ppm.

Models were re-run on the best features using different pre-preprocessing techniques with the dataset split 70:30 (train:test). The coefficient of determination of prediction, calibration and its respective root mean square errors for the prediction models are also displayed in the same table. The best calibration outcome for the prediction of TGA revealed an R^2^_p_ value of 0.671 and RMSE_p_ of 54.12 ppm upon the application of first derivative pre-treatment using 15 latent variables. Keeping the hyperparameters for SVMR constant, models were developed with the CARS + BE selected wavelengths. The best predicted results were observed upon applying the first derivative pre-treatment with an R^2^_p_ value of 0.649 and RMSE_p_ of 56.4 ppm. PLSR performed better than SVMR-based models using a reduced feature space. This could be because the predictor values (Y) inherently follow a linear pattern. Also, RBF-SVMR generally requires a larger dataset to recognize patterns in spectral data. With a smaller dataset such as ours, PLSR was able to reduce the data to a set of latent variables and display improved performance.

#### Variable Selection Using IRIV and BE

The IRIV process was able to reduce the feature space to an average of 23 from a total of 169 wavelengths. The best-performing PLSR model using the variables selected by IRIV was obtained upon the application of the first derivative operation. The prediction model delivered an R^2^_cv_ value of 0.571 and RMSE_cv_ of 64.10 ppm (Table 2). Backward elimination was applied to the IRIV-selected wavelengths, thereby further reducing the feature count to an average of 15, which is a 58% reduction in comparison to the CARS + BE outcome (Table 3). PLSR models developed using the LOOCV technique performed better than the SVMR-LOOCV models using the IRIV + BE-selected features. The application of |first derivative |+ SNV pre-treatment to develop the PLSR model predicted TGA the best, providing an R^2^_cv_ value of 0.634 and RMSE_cv_ of 59.20 ppm. The SVMR model developed upon applying the SNV + first derivative pre-treatment operation resulted in a slightly reduced metric of R^2^_cv_ = 0.624 and RMSE_cv_ = 60 ppm. (Table 5).

**Figure 5:**
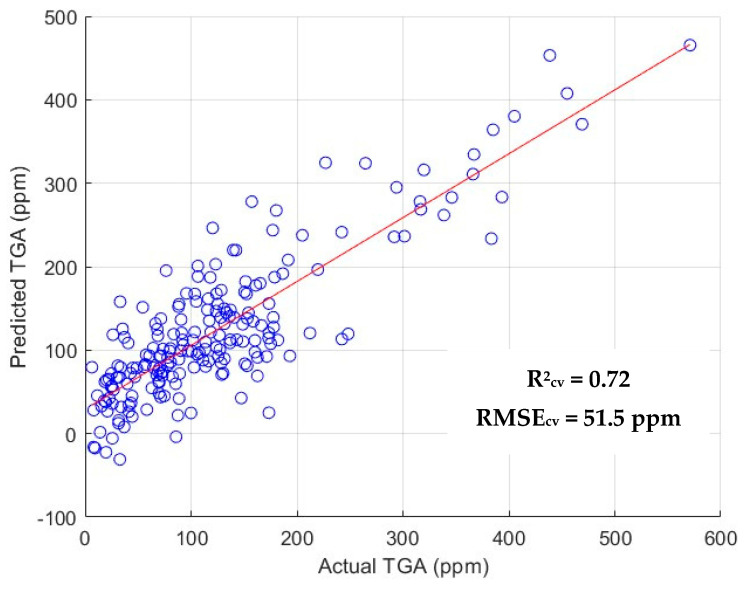
Scatter plot of predicted vs. actual TGA values for the best-performing model.

Model performances were further scrutinized by using a 70:30 (train:test) split resulting in metrics such as R^2^_p_, R^2^_c_, RMSE_p_ and RMSE_c_. Optimal PLSR model performance was observed upon the application of |first derivative |+ SNV pre-treatment, yielding an R^2^_p_ value of 0.584 and RMSE_p_ of 60.80 ppm. Among the SVMR models developed using IRIV + BE best features, the SNV + first derivative pre-treatment performed the best. Metrics indicated a R^2^_p_ value of 0.561 and RMSE_p_ of 63.30 ppm, which is a relatively poor performance compared to CARS-selected best features. It can be inferred from the RPD values generated by the PLSR and SVMR models that the CARS + BE feature selection technique performed better than its IRIV counterparts. An RPD value of 1.8 was achieved for the best predictive model, indicating that it is suitable for quantitative analysis, with room for improvement with a larger sample size. Such a condition might prevail because CARS was able to identify a higher number of critical wavelengths compared to IRIV. The application of BE to both CARS and IRIV enhanced model performance, which further indicates that useful wavelengths were retained, contributing to the development of a robust model. Comparing the performance of the full spectrum with those developed using CARS + BE and IRIV+ BE, it can be concluded that extraction of specific wavelengths improved the R^2^_cv_ and R^2^_p_. In summation, PLSR model with the application of first derivative pre-treatment performed best, resulting in an R^2^_cv_ of 0.723 and RMSE_cv_ of 51.50 ppm. The scatter plot of this model is pictorially represented in Figure 5.

It is worth noting that CARS and IRIV’s best features have eight wavelengths in common. The C-H first and second stretching overtones and its combination bands were captured by both feature selection techniques (1166.3, 1649.3, 1791.4, 2047.1, 2085.0 and 2378.5 nm), which largely comprise bands related to carbohydrates and lipids [30,31]. Since α-solanine and α-chaconine are connected to sugar moieties (as the name suggests), they are likely noticed by the hyperspectral imaging system as a combination of bands [32]. Additionally, a strong water region was also absorbed at 1933.4 nm, indicating the need for multivariate analysis (PLSR/SVMR) to estimate the total glycoalkaloid content in tubers.

Several non-destructive approaches have been taken to predict tuber physiochemical properties such as dry matter, sugar content and both external and internal defects due to their importance in the processing and grading industry. However, limited research specifically focuses on the prediction of TGAs, despite their classification as naturally occurring contaminants in potato tubers and the existence of regulatory thresholds. This highlights a critical gap in postharvest monitoring systems, particularly those aiming to ensure compliance with food safety standards.

A VIS-NIR system that could record 175 channels was used by Kjaer et al. [33] to concurrently predict the chlorophyll and TGA content in potatoes. R^2^ values were observed in the range of 0.2–0.3 with corresponding high RMSE values between 108 and 373 mg/kg, indicating limited predictive reliability. Another study by Tilahun et al. [34] attempted to predict α-solanine and α-chaconine in tubers using the VIS-NIR system and Hunter color readings as reference values. Coefficients of regression for the prediction of glycoalkaloids were in the 0.63–0.68 range, indicating the need for improved methodologies that can yield higher predictive accuracy.

Given the spectral limitations of the visible range, it can be inferred that extending the wavelength coverage into the short-wave infrared (SWIR) region significantly enhances the model’s predictive capacity. The present study demonstrates the potential of SWIR-based hyperspectral imaging not only as a tool for quality and process optimization, but also to support regulatory compliance by enabling accurate, non-destructive detection of natural toxins such as TGAs. By emphasizing the contaminant detection aspect, this research highlights how such technologies can play a critical role in improving both agricultural efficiency and food safety monitoring.

## 4. Conclusions

The study investigated the effective, non-destructive prediction of total glycoalkaloids (α-solanine and α-chaconine) in Yukon Gold potatoes. To develop the model, HPLC was used to quantify the actual TGA values in 210 light-exposed potatoes, to simulate greening. The concatenation of the actual values with the acquired spectral data, which provided critical insight into the functional groups and their vibrations in the SWIR range (900–2500 nm), helped develop the dataset. Signal pre-processing proved effective in the removal of background artifacts with derivative operations being the most useful. Since the highly dimensional full spectrum comprising 169 wavebands would result in slower model training, CARS and IRIV along with BE were used to select the most relevant features. The best-performing model was achieved by applying the first derivative pre-processing operation, on the CARS + BE best feature space, resulting in an R^2^_cv_ of 0.72 and RMSE_cv_ of 51.5 ppm. With a total of 26 wavelengths, this lab-scale proof of concept study can be scaled up to develop a multispectral system that can be automated and applied to sorting or grading equipment. Further implementation, however, would require a larger sample set that also considers variables such as variety and cultivar for enhanced prediction and robust model performance. In addition to improving efficiency in post-harvest processing, the accurate detection of TGAs addresses critical food safety concerns. By supporting compliance with established maximum recommended levels (MRL), such as Health Canada’s guideline of 200 mg/kg TGA (fresh weight), the method contributes to safer consumer products and improved quality control. Overall, the findings of this research contribute to the growing body of knowledge on sustainable and efficient post-harvest management practices, highlighting the role of SWIR hyperspectral imaging and machine learning in addressing key challenges in food safety and quality assurance.

## Figures and Tables

**Table 1:** PLSR and SVMR cross-validation results for TGA prediction using full spectrum (900–2500 nm).

Model	Pre-Processing	LV	R^2^_CV_	RMSE_cv_ (ppm)
	None	10	0.457	72.1
PLSR	SNV + 1st Derivative	10	0.366	77.9
	1st Derivative	10	0.366	77.9
				
	None	-	0.168	89.4
SVMR	SNV + 1st Der	-	0.417	74.7
	1st Derivative	-	0.418	74.6

**Table 2:** Cross-validation results of the PLSR model developed on CARS- and IRIV-selected features.

Model	Pre-Processing Technique	No. of Selected Wavelengths	LV	R^2^_CV_	RMSE_cv_ (%)	Wavelengths (nm)
CARS- PLSR	OSC + 1st Derivative	32	15	0.707	52.9	1166.3 1469.4 1478.8 1554.6 1649.3 1725.1 1753.5 1762.9 1791.4 1819.8 1848.2 1867.1 1876.6 1895.5 1914.5 1933.4 1971.3 2047.1 2075.5 2085 2141.8 2151.2 2179.7 2227 2236.5 2274.4 2283.8 2302.8 2312.2 2369.1 2378.5 2435.4
IRIV- PLSR	1st Derivative	23	15	0.571	64.1	1242.1 1403.1 1431.5 1469.4 1478.8 1583 1630.4 1649.3 1810.3 1819.8 1895.5 1933.4 1971.3 2009.2 2122.8 2141.8 2189.1 2255.4 2293.3 2312.2 2378.5 2416.4 2435.4

**Table 3:** Best features obtained upon performing BE on CARS- and IRIV-selected wavelengths.

Method	Pre-Processing Technique	No. of Selected Wavelengths	Wavelengths (nm)
	None	14	1033.7 1043.2 1062.1 1081.1 1128.4 1630.4 1829.2 1848.2 1857.7 2227 2293.3 2312.2 2388 2482.7
	SNV + 1st Derivative	31	1289.4 1403.1 1450.4 1469.4 1554.6 1649.3 1725.1 1753.5 1762.9 1819.8 1848.2 1867.1 1876.6 1895.5 1914.5 1971.3 2047.1 2075.5 2085 2132.3 2141.8 2151.2 2160.7 2236.5 2274.4 2283.8 2312.2 2369.1 2378.5 2435.4 2482.7
CARS	1st Derivative	31	1166.3 1450.4 1554.6 1649.3 1687.2 1725.1 1753.5 1762.9 1819.8 1848.2 1867.1 1876.6 1895.5 1914.5 1971.3 2047.1 2075.5 2085 2103.9 2141.8 2151.2 2179.7 2227 2236.5 2274.4 2283.8 2312.2 2331.2 2369.1 2378.5 2435.4
	OSC + 1st Derivative	26	1166.3 1469.4 1554.6 1649.3 1725.1 1753.5 1762.9 1819.8 1848.2 1867.1 1876.6 1895.5 1971.3 2047.1 2075.5 2085 2141.8 2151.2 2179.7 2227 2236.5 2274.4 2283.8 2369.1 2378.5 2435.4
			
	None	8	1431.5 1488.3 1781.9 1791.4 1810.3 1829.2 2227 2274.4
IRIV	SNV + 1st Derivative	14	1289.4 1469.4 1478.8 1630.4 1649.3 1810.3 1819.8 1971.3 2141.8 2151.2 2312.2 2369.1 2378.5 2435.4
	1st Derivative	16	1242.1 1403.1 1431.5 1469.4 1478.8 1583 1630.4 1649.3 1810.3 1819.8 1971.3 2141.8 2189.1 2312.2 2378.5 2435.4
	|1st derivative| + SNV	22	1033.7 1166.3 1223.1 1242.1 1289.4 1374.7 1412.5 1450.4 1649.3 1791.4 1933.4 1952.4 2047.1 2085 2122.8 2141.8 2160.7 2198.6 2293.3 2350.1 2378.5 2388

**Table 4:** PLSR and SVMR model metrics using CARS best features.

Model	Pre-Processing	R^2^_CV_	RMSE_cv_ (ppm)	R^2^_C_	RMSE_c_ (ppm)	R^2^_p_	RMSE_p_ (ppm)	RPD
	None	0.603	61.7	0.67	55.8	0.568	62.8	1.57
	SNV + 1st Derivative	0.714	52.3	0.817	41.55	0.661	54.82	1.79
PLSR	1st Derivative	0.723	51.5	0.822	40.84	0.671	54.12	1.81
	OSC + 1st derivative	0.721	51.7	0.824	40.88	0.642	40.88	1.76
								
	None	0.478	70.7	0.791	44.4	0.227	80.96	1.21
	SNV+ 1st Der	0.698	53.7	0.807	42.63	0.618	58.55	1.67
SVMR	1st Derivative	0.693	54.2	0.764	47.1	0.649	56.41	1.73
	OSC+ 1st derivative	0.711	52.6	0.768	46.6	0.644	57.10	1.71

**Table 5:** PLSR and SVMR model metrics using IRIV best features.

Model	Pre-Processing	R^2^_CV_	RMSE_cv_ (ppm)	R^2^_C_	RMSE_c_ (ppm)	R^2^_p_	RMSE_p_ (ppm)	RPD
	None	0.481	70.5	0.537	66.3	0.444	70.7	1.39
	SNV + 1st Derivative	0.598	62.0	0.674	55.6	0.553	62.9	1.56
PLSR	1st Derivative	0.60	61.9	0.688	54.45	0.554	62.9	1.56
	|1st Der| + SNV	0.634	59.2	0.740	49.59	0.584	60.8	1.61
								
	None	0.498	69.4	0.574	63.6	0.469	69.5	1.41
	SNV+ 1st Der	0.624	60	0.658	56.7	0.561	63.33	1.54
SVMR	1st Derivative	0.587	62.9	0.654	57.3	0.563	62.48	1.57
	OSC+ 1st derivative	0.583	63.2	0.670	55.8	0.552	63.90	1.53

## Data Availability

The original contributions presented in this study are included in the article. Further inquiries can be directed to the corresponding author.
